# Urine Nitric Oxide Is Lower in Parents of Autistic Children

**DOI:** 10.3389/fpsyt.2021.607191

**Published:** 2021-05-21

**Authors:** Lulu Yao, Kun Cai, Fanghua Mei, Xiaohua Wang, Chuangang Fan, Hong Jiang, Fang Xie, Ying Li, Lu Bai, Kang Peng, Wenwen Deng, Shenghan Lai, Jun Wang

**Affiliations:** ^1^National 111 Center for Cellular Regulation and Molecular Pharmaceutics, Hubei University of Technology, Wuhan, China; ^2^Department of Biomedicine and Biopharmacology, Hubei University of Technology, Wuhan, China; ^3^Hubei Provincial Center for Disease Control and Prevention, Wuhan, China; ^4^School of Social Development and Public Policy, Beijing Normal University, Beijing, China; ^5^Department of Child Health Care, Huangshi Maternity and Child Health Care Hospital, Wuhan, China; ^6^Department of Pathology, Johns Hopkins University School of Medicine, Baltimore, MD, United States

**Keywords:** autism spectrum disorder, parents, nitric oxide, nitrite, nitrate, urine

## Abstract

Parents raising children with autism spectrum disorder (ASD) usually carry on their daily life under tremendous stress, but limited empirical research has been devoted to this population. It is known that parents' health status directly impacts therapeutic outcome of ASD children. As an important regulator in cardiovascular, nervous and immune systems, nitric oxide (NO) levels haven't been reported in parents of ASD children yet. In this study, we measured urine nitrite and nitrate from 43 ASD parents (ASD-P), and 43 healthy adults in the same range of age (Control) who didn't have any ASD descendants. Comparison between the ASD-P and Control groups showed that ${\text{NO}}_{2}^{-}$, ${\text{NO}}_{3}^{-}$, and ${\text{NO}}_{2}^{-}$/${\text{NO}}_{3}^{-}$ were all significantly lower in the ASD-P group. Analysis on the interaction effect of sex and group indicated that urine ${\text{NO}}_{3}^{-}$ of mothers in ASD-P was lower than that in females of the Control group, but no significant difference was observed between males in both groups. It is for the first time that urine nitric oxide metabolites (nitrite, nitrate) levels were precisely reported to differentiate parents of autistic children from other adults without ASD descendants. This phenomenon suggests that parents (especially mothers) of autistic children might have experienced more mental and physical stressors, which led to decreased NO levels during metabolism. Further investigations are necessary to uncover the etiology of low urine NO among parents of autistic children.

## Introduction

Autism spectrum disorder (ASD) is a severe neurological developmental disease, negatively impacting millions of children and their families worldwide (1). Along with better diagnostic tools, more and more children have been identified with this disorder, with the prevalence about 1 out 100 in China (2). Research has shown that participation of parents was critical for improvement of core ASD symptoms of these children, which was dependent on the health status of their parents (3). Very limited empirical studies have focused on pathophysiological research of ASD children' parents (ASD-P), though research on ASD children has been extensive. In fact, ASD-P are usually under chronic stress. For example, due to financial strain, intense caregiving demands and other related stressors, these parents generally exhibited elevated levels of stress, anxiety and depression than the general public, which was strongly associated with the core symptoms and behavioral problems of their autistic children (4, 5). In addition, mothers were usually more prone to depression compared to fathers of ASD children (5). Stress not only causes psychological problems, but also leads to cardiovascular diseases, depending on the degree and duration of stress, as well as individuals' responses (6).

Impairment of nitric oxide (NO) synthesis or bioactivity has been known to be an independent risk factor for cardiovascular dysfunction. Decreased NO was observed in plasma and urine of patients with cardiovascular disorders (7). Other than the role of vasodilator, NO also serves as a mediator in the nervous system. It regulates neuron's survival, proliferation and differentiation. NO also mediates synaptic activity, plasticity and vesicle trafficking, including regulation of important neurotransmitters such as serotonin, dopamine and glutamate (8). Low plasma NO levels were reported in adults with depression (9). Decreased L-arginine level was found in adults with major depressive disorder (10), which is the substrate of NO synthesis under normoxia. Besides, dysfunction of nitric oxide synthases (NOSs) was reported in patients with psychological problems, including endothelial nitric oxide synthase (eNOS) and neuronal nitric oxide synthase (nNOS) in prefrontal cortex (11), platelet eNOS activity or levels of plasma NO metabolites (NO_x_) (12), density of NOS in immunoreactive neurons in the paraventricular nucleus (13) and nNOS immunoreactivity in the locus coeruleus (14). However, contradictive findings were also noted in available literatures. Similar or elevated NO in blood was observed in patients with depression, with plasma nitrate returned approximately to the control levels upon recovery (15, 16). Conflicting results were reported from various studies on the function of NO in psychological disorders. The role and pathways of NO are complicated and deserves further investigation.

Previous studies suggested that sex, age, and ethnicity might affect NO production. One study comparing serum NO_x_ levels of healthy individuals at different ages reported that NO_x_ levels were significantly higher in men aged 20–29 years than in women, and the serum NO_x_ reached the peak at 50–59 years old for both men and women (17). In one study of exhaled NO, it was found that male NO levels were significantly higher than those in females, and Chinese children had significantly greater exhaled NO levels than white children, independent of age (18, 19). In the study of periodontitis disease, saliva ${\text{NO}}_{2}^{-}$ and serum NO_x_ levels in male participants with periodontitis were significantly lower than those in the control group, but there was no significant difference in female participants between the two groups (20). In animal experiments, the basal release of endothelial-derived nitric oxide (EDNO) in aorta of wild-type male mice was significantly higher than that of wild-type female mice, while that of male estrogen receptor knockout (ERKO) mice was significantly lower than that of male wild-type mice, suggesting that the basal release of EDNO in aorta of mice was related to estrogen receptor, and it was speculated that the observed decrease of vascular estrogen receptor number might be a new risk factor for cardiovascular disease (21). In another study, the levels of eNOS mRNA and protein in the kidney tissue of female rats were 80% higher than those of male rats (22). NO and these endogenous molecules are influenced by sex, age, and probably ethnicity. Therefore, it is not surprising that different studies in human groups and animal models have shown conflicting results. In short, further studies are necessary to clarify the controversial results about roles of sex and age in NO production.

To date and to our knowledge, NO levels in parents of autistic children (ASD-P) have not been reported in the available literature. As the main caregivers of ASD children, ASD-P often suffer more economic, psychological and physical pressure than other parents. Their psychological and physical health deserves more attention. In order to understand the pathophysiological status and evaluate NO bioavailability in this specific population, we measured urinary nitrite and nitrate from 43 parents of autistic children (ASD-P) in this study, who were not under any medication. In comparison, 43 healthy adults were recruited as the Control group, who didn't have any ASD descendants. The excretion of creatinine reflects kidney function, and the daily creatinine production of human beings is usually constant. Here creatinine was quantified to adjust the levels of urinary nitrite and nitrate (NO_x_). Comparison of urine NO_x_ between these two groups was performed, as well as between males and females in each group. Correlation between NO_x_ and age was also investigated. In order to strengthen the findings of NO in urine, we also measured serum NO_x_ from 38 of the ASD-P group and another group of healthy adults (40 people, S-Control). Since some participants in the Control group worked in another city or were under medication during blood sampling, the S-Control group was composed of newly recruited healthy volunteers who lived in Wuhan City. In order to rule out the bias of food intake of NO_x_, we also measured nitrite and nitrate from three types of most common and affordable food (spinach, banana and grass carp) eaten by these parents of autistic children, which were obtained from three booths randomly at two biggest markets in the region where the ASD-P group lived.

## Materials and Methods

### Subject Recruitment

Forty three parents of autistic children (ASD-P) and 43 healthy adults (Control), who didn't have any ASD descendants, were recruited for analysis of NO_x_ in urine. Then, we re-recruited 40 healthy adults (S-Control) and 38 parents of these autistic children for serum NO_x_ analysis. All the volunteers were recruited at Huangshi Maternity and Child Health Hospital (Wuhan City, Hubei Province, China) and none of these participants was under medication. The regulation policy on human subject research was followed. Urine and serum were obtained, processed and analyzed in compliance with national guidelines in submission of this manuscript. Biomedical consent and protocols had been reviewed and approved by the Ethics Committee of Huangshi Maternity and Children's Health Hospital (case number: 2017-KF-001).

### Urine Sampling

Spot urine specimens of all the participants were collected using a one-time propene polymer urine cup at 8:30 am after overnight fasting. Urine was then transferred into a 10 mL propene polymer tube for immediate analysis or storage at −80°C for next-step management.

### Pretreatment of Urinary Samples

For creatinine analysis, urine specimens were centrifuged at 3,000 rpm for 3 min, and then supernatant was diluted 10 times for detection. For nitrite and nitrate analysis, fresh or thaw-on-ice urine samples were directly injected into the purge vessel of Nitric Oxide Analyzer (NOA) 280i for quantitation, unless dilution was needed (23).

### Measurement of Urine Creatinine

Urine creatinine was measured via the Creatininase HMMPS Method (24). It was quantified by measuring absorbance of a blue pigment of oxidation condensation with N-(3-sulfopropyl)-3-methoxy-5-methylaniline (HMMPS) and 4-aminoantipyrine (4-AA).

After urine was collected, it was centrifuged at 3,000 rpm for 3 min, and the supernatant was diluted 10 times with ultra-pure Milli-Q water. Then 200 μL diluted supernatant was treated with reagent R1 and R2 according to instruction of the Creatininase HMMPS kit (Lot # 19032705, Beijing Leadman Biochemistry Co., China). Creatinine concentration was calculated and reported via measuring the final product of a blue pigment at 600 nm on Leadman Automatic Biochemical Analyzer AU480 (Beijing Leadman Biochemistry Co., China).

### Measurement of Urinary Nitrite and Nitrate

Nitrite and nitrate were detected with Nitric Oxide Analyzer (NOA 280i, GE, USA) with nitrogen as the carrier gas. Reagent of 0.011 g/mL iodine ion was applied for nitrite detection and 0.008 g/mL acidic vanadium trichloride solution was used for nitrate detection. The standard curve was generated via measurement of duplicate injections of each standard solution with *R*^2^ > 0.999. Each urine specimen was injected three times with the volume of 100 μL (25, 26).

### Statistical Analysis

A two-tailed unpaired Student's *t*-test was performed to compare significant difference of NO_x_ level between different groups such as ASD-P vs. Control, ASD-P vs. S-Control, and male vs. female. Pearson's Correlation was performed to analyze the correlation between age (gender) and NOx level by Graph Pad Prism 7.0 (Graphpad, San Diego, California, USA). Multivariate analysis of variance (MANOVA) was performed to explore the interaction of multiple independent variables (including gender, group and age group) on the NO_x_ level in urine and serum by SPSS 22.0 (IBM, Armonk, New York, USA), the sex and group interaction on NO_x_ were verified by ANOVA with SPSS 22.0. Values for all measurements were expressed as mean ± SD for parametric distributions. *P* < 0.05 was considered statistically significant. All experiments were performed at least three times.

## Results

### Subjects Recruited for This Study

Among 43 recruited parents of ASD children (ASD-P), 19 fathers and 24 mothers were 20–50 years old. They were the major caregivers for their children who had been taking intervention or therapy due to clinical autistic symptoms for at least 6 months at Huangshi Maternity and Child Health Hospital (Wuhan City, Hubei Province, China). Diagnosis of ASD children was conducted by two independent professional clinicians from Huangshi Maternity and Child Health Care Hospital, primarily based on the Diagnostic and Statistical Manual of Mental Disorders (Fifth Edition, DSM-5). The Autism Diagnostic Observation Schedule (ADOS) was used as a diagnostic aid. Exclusion criteria were parents of children who diagnosed with other psychological problems (e.g., attention deficit hyperactivity disorder, obsessive-compulsive disorder), a history of brain injury, severe chronic conditions (e.g., gastrointestinal symptoms), definite genetic metabolic syndrome, recent infections, recent high-doses of vitamin or mineral supplements, and any medication. The Control group had 43 healthy adults, including 16 male and 27 female, who didn't have any autistic descendants or children with other psychological problems. In order to explore whether NO levels in both blood and urine samples share the similar trend, later on we collected blood specimens from 38 out of the 43 originally recruited parents of ASD children (ASD-P), as well as from another group of 40 healthy adults (S-Control) who didn't have any autistic descendants or children with other psychological problems. This S-Control group is newly recruited population, for some people of the Control group either worked in another city or were currently under medication. Serum NO_x_ were measured in 38 ASD-P (18 fathers and 20 mothers), 40 S-Control adults (20 men and 20 women). All participants agreed with the research consent, and no one was under medication.

### Validation of NOA 280i for Measurement of Nitrite and Nitrate

To verify the reliability of the chemiluminescence method to measure nitrite and nitrate, standard solutions of nitrite and nitrate were prepared in ultra-pure Milli-Q water with sodium nitrite and sodium nitrate as standard chemical compounds. Nitrite and nitrate were detected using NOA 280i (GE, US). Nominal concentration was set at 1.0, 2.0, 3.0, 5.0, 8.0, and 10.0 μM for nitrite, and 1.0, 2.0, 3.0, 5.0, 8.0, and 10.0 μM for nitrate. A diagram of area under curve (AUC) vs. theoretical NO amount (picomole, pmol) were created to evaluate the daily reliability of NOA 280i. The slope of the fitted line falls into the range 0.09–0.13, i.e., the value set by manufacturer, which means that NOA 280i passes the daily test and is ready for use.

The results (Supplementary Figure 1) showed that AUC fitted well with the theoretical amount of nitrite and nitrate, with slope equal to 0.1053 for nitrite (units of area/pmol, *R*^2^ = 0.9999) and 0.1013 (units of area/pmol, *R*^2^ = 0.9993) for nitrate, respectively. These results confirmed the reliability of this chemiluminescent method for NO_x_ analysis.

### Creatinine in the ASD-P and Control Groups

**Scheme 1:**
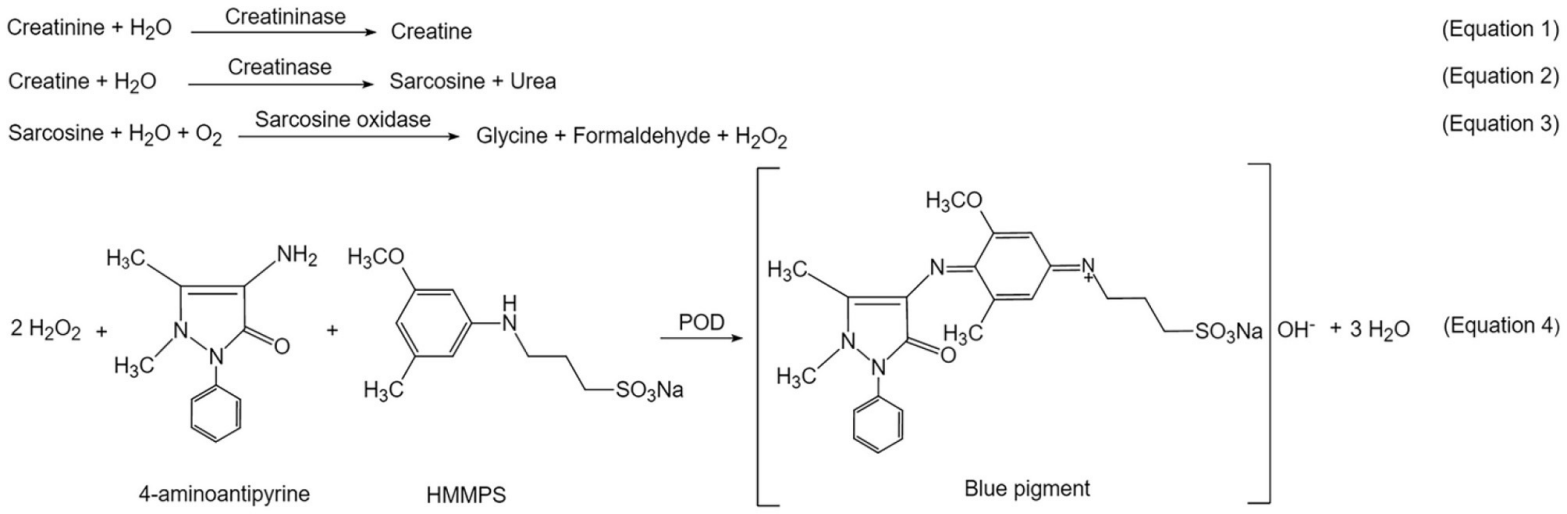
The creatininase HMMPS method for creatinine quantification.

Creatinine is a marker of glomerular function. It is commonly used for adjustment of urinary analytes. The level of urinary creatinine hasn't been reported in the ASD-P population yet. In this study, urine creatinine was quantified for parallel comparison between the ASD-P and Control groups, as well as for adjustment of urine NO_x_. By using a commercial creatininase HMMPS kit, creatinine was first converted to creatine in the presence of creatininase (Equation 1, Scheme 1). The latter was hydrolyzed to generate sarcosine and urea (Equation 2). Then sarcosine oxidase catalyzed sarcosine decomposition to form hydrogen peroxide (Equation 3), which triggered oxidative condensation of N-(3-sulfopropyl)-3-methoxy-5-methylaniline (HMMPS) and 4-aminoantipyrine (4-AA) to produce a blue pigment with maximum absorbance at 600 nm (Equation 4) (27).

**Figure 1:**
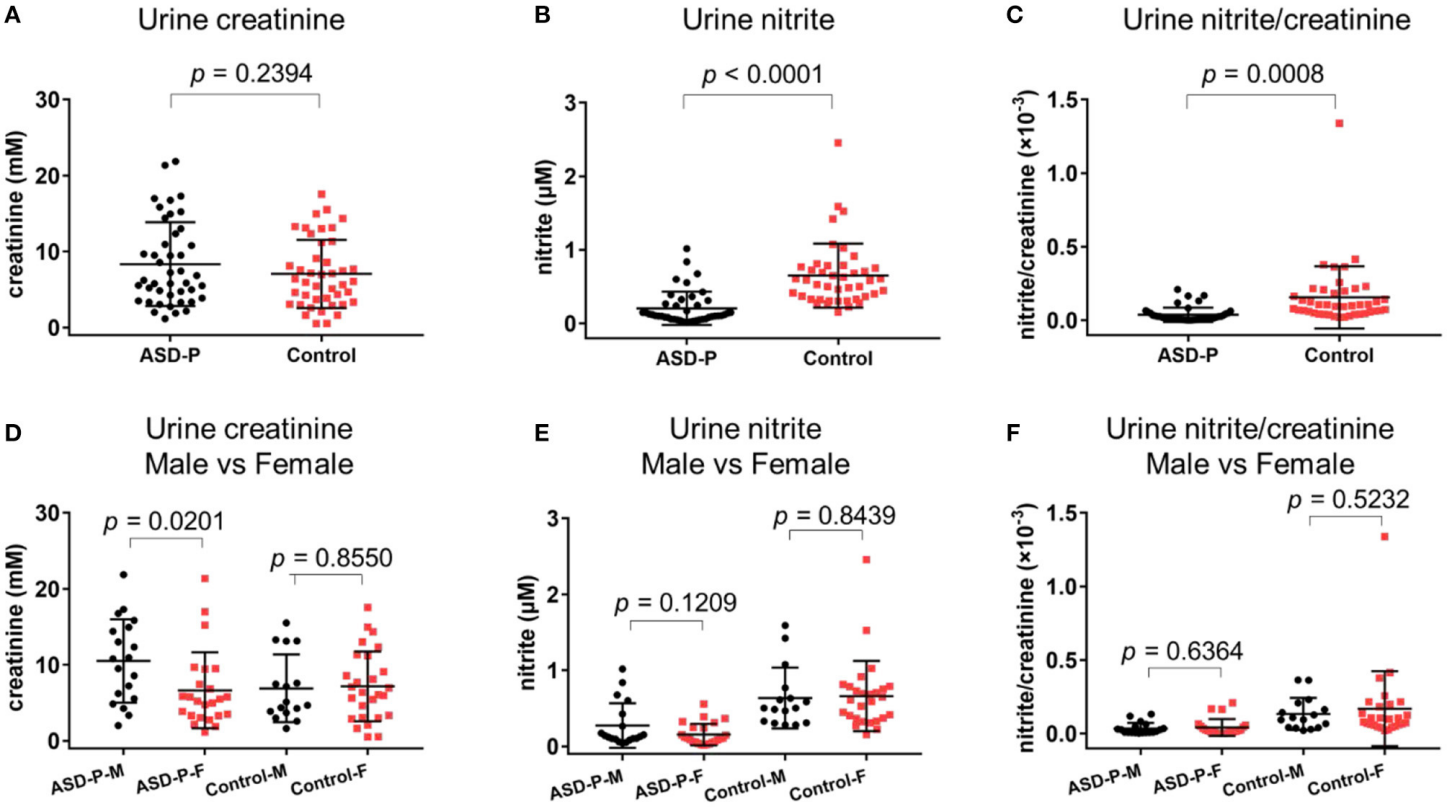
Levels of urine creatinine, nitrite, nitrite/creatinine in the ASD-P and Control groups. **(A)** creatinine concentrations in two groups. **(B)** nitrite concentrations in two groups. **(C)** nitrite/creatinine levels in two groups. **(D)** creatinine concentrations for male and female participants. **(E)** nitrite concentrations for male and female participants. **(F)** nitrite/creatinine levels for male and female participants.

**Figure 2:**
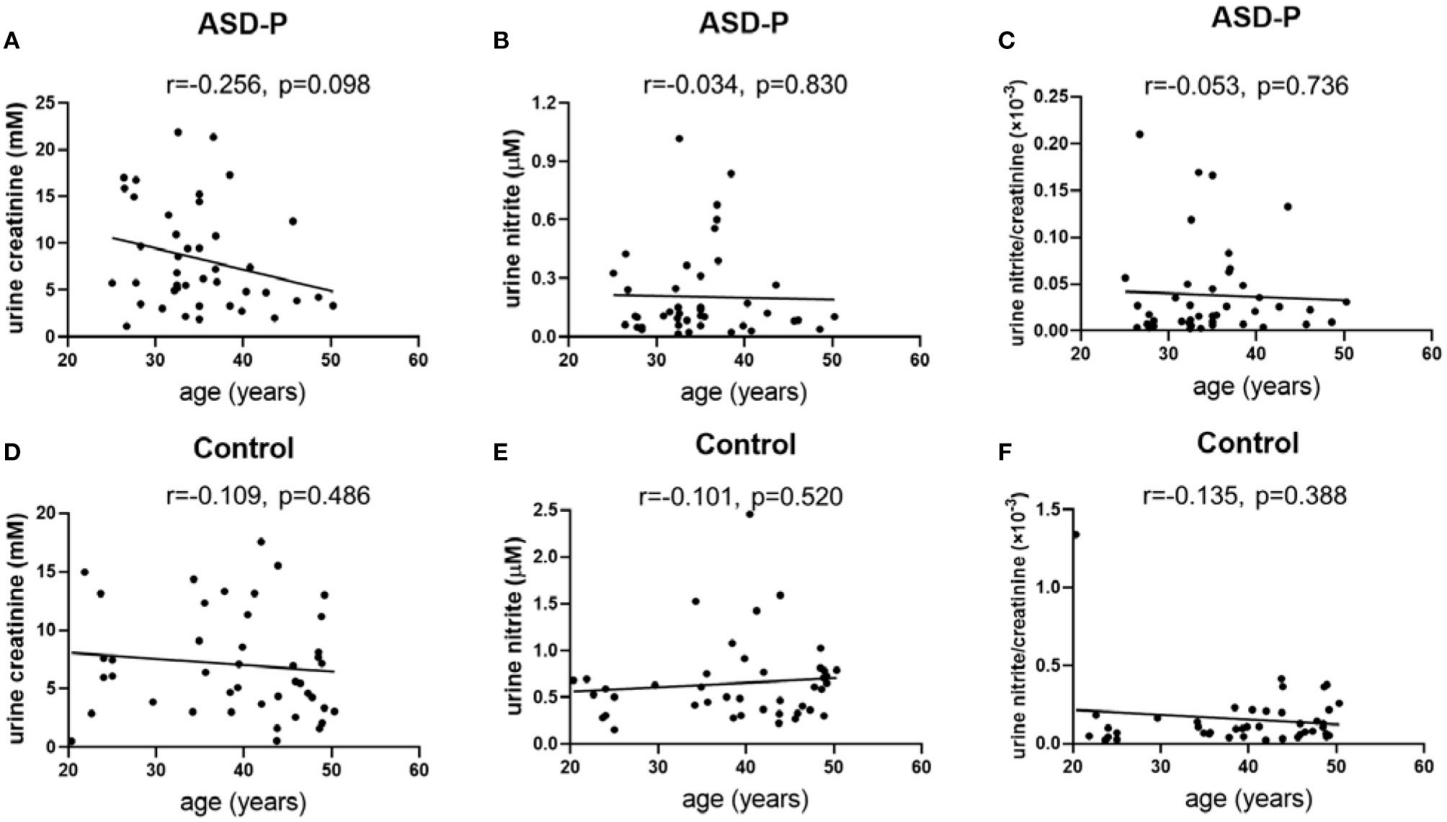
Examination of urine creatinine, nitrite and nitrite/creatinine vs. age. **(A)** urine creatinine and age in the ASD-P group, *p* = 0.098. **(B)** urine nitrite and age in the ASD-P group, *p* = 0.830. **(C)** urine nitrite/creatinine and age in the ASD-P group, *p* = 0.736. **(D)** urine creatinine and age in the Control group, *p* = 0.486. **(E)** urine nitrite and age in the Control group, *p* = 0.520. **(F)** urine nitrite/creatinine and age in the Control group, *p* = 0.388.

The results with Student's *t*-test analysis showed that no significant difference of urine creatinine levels was found between the ASD-P and Control groups (Figure 1A), with the average levels of creatinine equal to 8.352 ± 5.513 mM (1.15–21.87 mM) for ASD-P, 7.067 ± 4.487 mM (0.51–17.55 mM) for the Control, respectively. As for comparison between male and female volunteers in each group, male subjects had significantly higher levels of creatinine than females in the ASD-P group (*p* = 0.0201), with no significant difference inside the Control group (*p* = 0.8550) (Figure 1D). Neither multivariate analysis of variance (MANOVA) nor one-way analysis of variance (ANOVA) after adjustment of age and sex showed any sex and group interaction on creatinine. Furthermore, Pearson's correlation analysis showed that no correlation between age and creatinine was observed in the ASD-P and Control groups (Figures 2A,D). Afterwards, creatinine was applied for adjustment of urinary ingredients NO_x_ in this study.

### Urine Nitrite in the ASD-P and Control Groups

Spot urine was collected after overnight fasting, and then was immediately transferred to the lab and was quantified there with NOA 280i. Student's *t*-test was applied for comparison between two groups. Among 43 parents, the nitrite level was 0.2068 ± 0.2277 μM, in the range of 0.0151–1.0173 μM. Among the Control group, the nitrite level was 0.6518 ± 0.4355 μM (0.155–2.4577 μM). Obviously, nitrite in ASD-P was significantly lower than that in Control group, with *p* < 0.0001 (Figure 1B, Table 1). After normalization with creatinine, effect remained significant, with p equal to 0.0008 for nitrite/creatinine between the ASD-P and the Control (Figure 1C, Table 1, Supplementary Table 1).

**Table 1:** Summary of urinary NO_x_, NO_x_/CREA, and ${\text{NO}}_{2}^{-}$/${\text{NO}}_{3}^{-}$ among adults.

**Groups**	**Number of cases/controls**	**Method**	**Finding compared to control**	**References**
ASD-P vs. Control	43/43	Chemiluminescence	${\text{NO}}_{2}^{-}$:0.2068 ± 0.2277 vs. 0.6518 ± 0.4355 μM^*^^a^ ${\text{NO}}_{3}^{-}$:3.142 ± 2.144 vs. 4.627 ± 3.847 mM^*^^a^ ${\text{NO}}_{2}^{-}$/CREA: 0.0383 ± 0.0497vs. 0.1564 ± 0.2114 × 10^−3^^*^^a^ ${\text{NO}}_{3}^{-}$/CREA: 0.5208 ± 0.4755 vs. 0.7986 ± 0.7876^*^^a^ ${\text{NO}}_{2}^{-}$/${\text{NO}}_{3}^{-}$: 0.0789 ± 0.0703 × 10^−3^ vs. 0.3476 ± 0.6208 × 10^−3^^*^^a^	This work
Hypertension vs. Control	19/11	GC-MS	NO_X_/CREA: 56 ± 17 vs. 77 ± 23 μmol/mmol^*^^a^	(49)
Hypertension vs. Control	9/12	Chemiluminescence	NO_X_/CREA: 42 ± 6 vs. 62 ± 7μmol/mmol^*^^a^	(50)
Hypertension vs. Control	8/8	GC-MS	${\text{NO}}_{3}^{-}$/CREA: 102.9 ± 18.1 vs. 183.4 ± 27.2 μmol/mmol^*^^b^	(51)
Alcoholic liver and hypertension vs. Control	10/10	IC-MS	NO_X_/CREA:42.8 ± 8.6 vs. 62.5 ± 11.5 μmol/mmol^b^	(52)
Diabetic vs. Control	30/20	Griess reaction	${\text{NO}}_{2}^{-}$:13.61 vs. 11.02 μM^c^	(53)
Hypertensive pregnant women vs. Control	43/40	chemiluminescence	${\text{NO}}_{2}^{-}$/${\text{NO}}_{3}^{-}$: 0.3 ± 0.2 ×10^−3^ vs. 0.6 ± 0.3 ×10^−3^^*^^a^	(54)
Growth hormone Deficiency patients vs. Control	30/30	GC-MS	${\text{NO}}_{3}^{-}$/CREA:96.8 ± 7.4 vs. 167.3 ± 7.5 μmol/mmol^*^^b^	(55)
Migraine patients vs. Control	30/20	Griess reaction	NO_X_/CREA:0.77 ± 0.14 vs. 0.28 ± 0.15 mmol/mmol^*^^a^	(56)
Hypertension vs. Control	13/25	HPLC	NO_X_: 126.0 ± 22.9 vs. 199.5 ± 20.6 mg/L^*^^a^	(33)
UTI vs. Control	73/241	LC-MS/MS	${\text{NO}}_{2}^{-}$:15.2 ± 65 vs. 0.89 ± 0.86 μM^*^^a^ ${\text{NO}}_{3}^{-}$:0.36 ± 0.32 vs. 1.89 ± 1.71 mM^*^^a^	(57)

* *p < 0.05*.

a *mean ± SD*,

b *mean ± SEM*,

c *mean*.

*CREA, creatinine; UTI, urinary tract infection patients*.

Afterwards, the sex factor was analyzed to see if it impacted urinary nitrite. No significant difference was observed between males and females in both groups (Figures 1E,F). Sex and group interaction wasn't found on urine nitrite, either. After adjustment with sex and age, ANOVA analysis didn't show any interaction effect. In addition, the correlation between age and nitrite was missing in both groups (Figures 2B,C,E,F), which indicates that age didn't play a role in nitrite production in the range of 20–50 years old.

### Serum Nitrite in the ASD-P and S-Control Groups

After overnight fasting, blood was collected into a tube without anticoagulant. After centrifugation, the upper-layer serum was transferred to an Eppendorf tube, which was immediately translocated to the lab and was quantified with NOA 280i. Student's *t*-test was applied for comparison between two groups.

The results (Supplementary Figure 2, Supplementary Table 2) showed that serum nitrite in the ASD-P group was also significantly lower than that in the S-Control group (*p* < 0.0001), with 0.3167 ± 0.2304 μM (0.0577–1.111 μM) and 0.6155 ± 0.3431 μM (0.0624–1.552 μM), respectively. Sex didn't make a difference for serum nitrite in the ASD-P group, but it indicated healthy women had higher nitrite levels (Supplementary Figure 2D). Additionally, we found sex and group interaction on serum nitrite with MANOVA. A simple effect analysis showed that serum nitrite in females of the ASD-P group was significantly lower than that in the S-Control group, with 0.3056 ± 0.2760 μM and 0.7935 ± 0.2863 μM (*p* = 0.000), respectively (Supplementary Figure 3C). The mean nitrite level of male participants in the ASD-P group was not significantly different from that in the S-Control group, with 0.3291 ± 0.1734 μM and 0.4375 ± 0.3049 μM (*p* = 0.193), respectively (Supplementary Figure 3C).

### Urine Nitrate in the ASD-P and Control Groups

**Figure 3:**
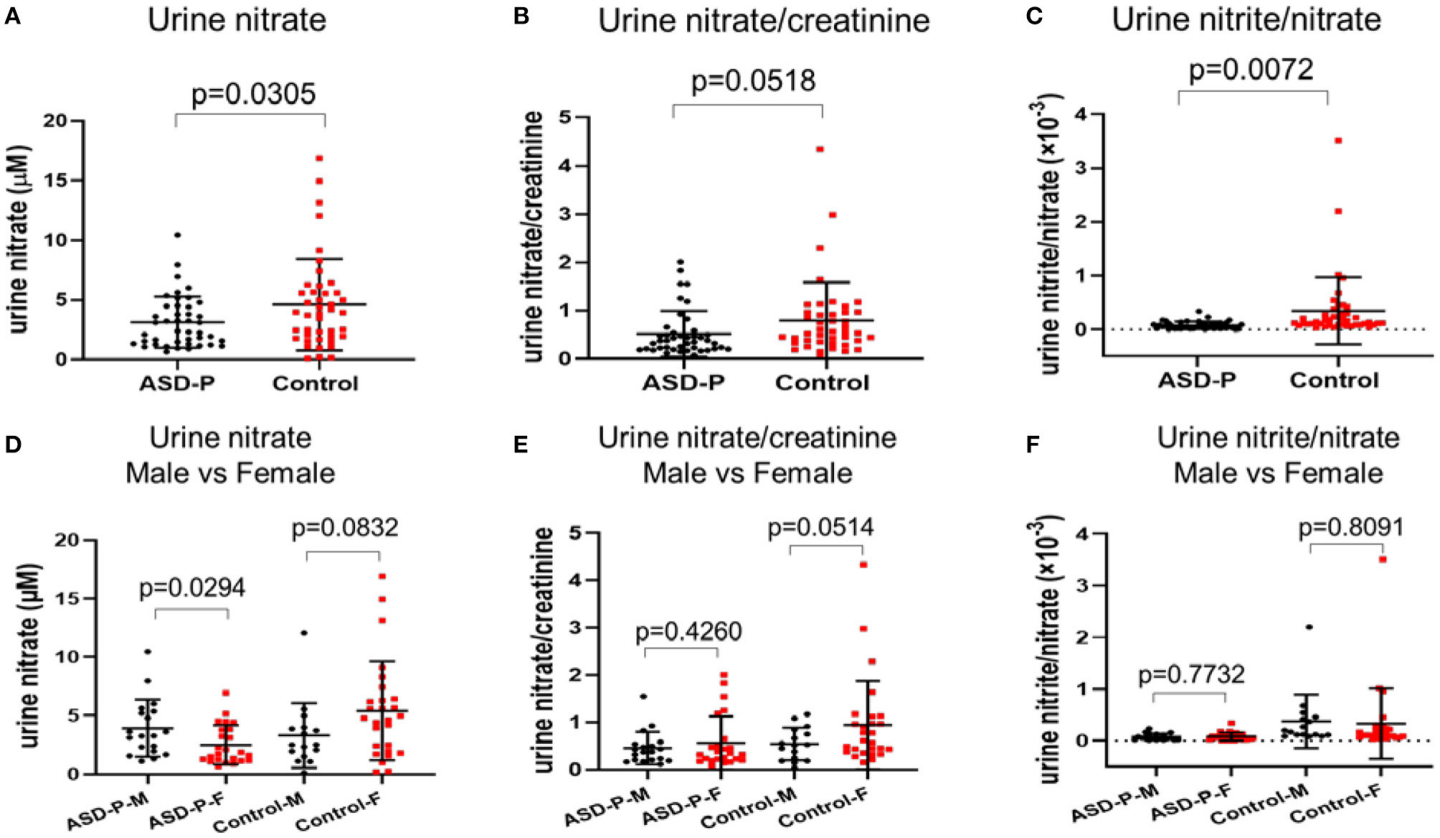
Levels of urine nitrate, nitrate/creatinine and nitrite/nitrate in the ASD-P and Control groups. **(A)** urine nitrate concentrations in two groups. **(B)** urine nitrate/creatinine levels in two groups. **(C)** urine nitrite/nitrate levels in two groups. **(D)** urine nitrate for male and female participants in each group. **(E)** urine nitrate/creatinine for male and female participants in each group. **(F)** urine nitrite/nitrate for male and female participants in each group.

The reductive VCl_3_ method was applied to measure the sum of urine nitrite and nitrate. nitrate concentration were obtained after subtracting nitrite. Results with Student's *t*-test analysis showed that nitrate levels were 3.142 ± 2.144 mM (0.6615–10.4814 mM) for ASD-P, and 4.627 ± 3.847 mM (0.1276–16.9125 mM) for the Control, with *p* = 0.0305 (Figure 3A, Table 1). After adjustment with creatinine, the trend remained with marginal difference (Figure 3B, Table 1). In each group, nitrate levels between males and females were compared. The results (Figures 3D,E, Table 1) indicated that mothers had lower nitrate than fathers in the ASD-P group before creatinine adjustment, and women had higher nitrate than men in the Control group. Then the ratio of nitrite/nitrate was analyzed in both groups, which was significantly higher in the Control group, with *p* = 0.0072 (Figure 3C). When the sex factor was considered, it showed no difference between males and females inside each group (Figure 3F).

**Figure 4:**
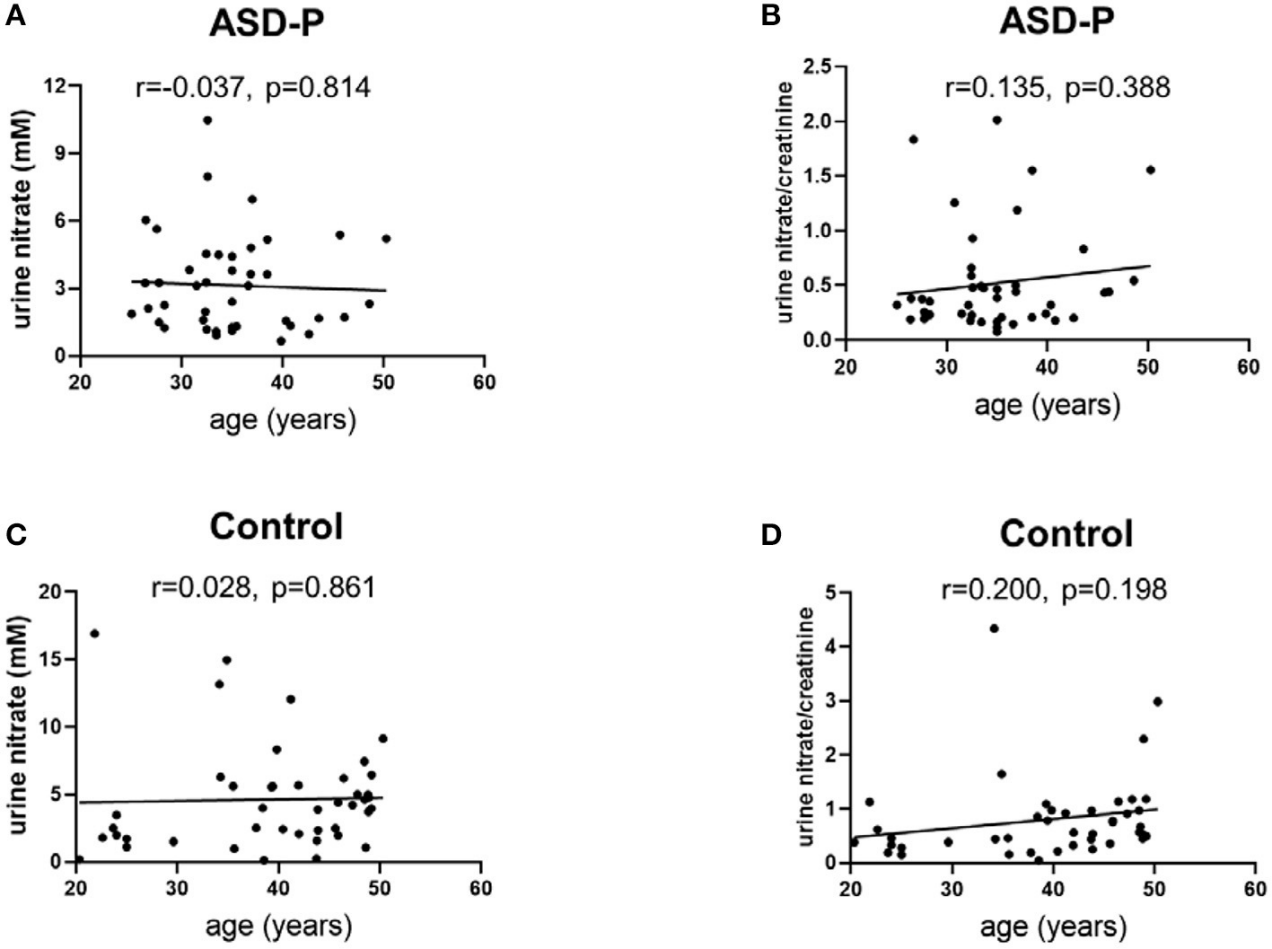
Examination of urine nitrate and nitrate/creatinine vs. age. **(A)** urine nitrate and age in the ASD-P group, *p* = 0.814. **(B)** urine nitrate/creatinine and age in the ASD-P group, *p* = 0.388. **(C)** urine nitrate and age in the Control group, *p* = 0.861. **(D)** urine nitrate/creatinine and age in the Control group, *p* = 0.198.

MANOVA analysis showed that there was significant sex and group interaction on nitrate (Supplementary Figure 3A). A simple effect analysis showed that nitrate in mothers of the ASD-P group was significantly lower than that in the Control group, with 2.514 ± 1.676 mM and 5.409 ± 4.218 mM (*p* = 0.002) (Supplementary Figure 3A), respectively. The mean nitrate level of fathers in the ASD-P group was not significantly different from that in men of the Control group, with 3.934 ± 2.44 mM and 3.308 ± 2.763 mM (*p* = 0.481), respectively (Supplementary Figure 3A). After adjustment with creatinine, nitrate/creatinine levels between females from two groups was borderline-significant, with 0.5703 ± 0.5601 in the ASD-P group and 0.9479 ± 0.9336 in the Control group (*p* = 0.091), respectively (Supplementary Figure 3B). No significant difference was found between males from two groups before or after adjustment with creatinine. This interaction effect of sex and group suggests that mothers of autistic children might experience more stressors that caused lower nitrate in urine. Examination of age effect on nitrate or nitrate/creatinine levels showed that age didn't play a role in urinary nitrate production for both groups, at least in the range of 20–50 years old (Figures 4A–D).

### Serum Nitrate in the ASD-P and S-Control Groups

Serum nitrate was also quantified via NOA 280i with VCl_3_ as the reductant. Student's *t*-test analysis showed there was no significant difference of serum nitrate levels between two groups, with 39.94 ± 22.88 μM (12.11–109.1 μM) in the ASD-P group and 46.22 ± 26.36 μM (5.355–115.4 μM) in the S-Control group (*p* = 0.2658), respectively (Supplementary Figure 2B). When nitrite/nitrate was compared, the S-Control group had significantly higher nitrite/nitrate levels than the ASD-P group (Supplementary Figure 2C). In each group, serum nitrate and nitrite/nitrate trends were slightly different between males and females (Supplementary Figures 2E,F). Similar to what was observed in serum nitrite, MANOVA analysis also showed that there was significant sex and group interaction on female serum nitrate levels (Supplementary Figure 3D). A simple effect analysis showed that serum nitrate in females of the ASD-P group was significantly lower than that in the S-Control group, with 27.60 ± 13.49 μM and 46.19 ± 27.56 μM (*p* = 0.011), respectively. There was no significant difference of serum nitrate found between males from two groups.

Furthermore, Pearson's correlation analysis showed that there was no correlation between age and serum NO_x_ in the ASD-P and S-Control groups (Supplementary Figures 4A–F), except between serum nitrite and age in the S-Control group (Supplementary Figure 4D). Furthermore, we did correlation analysis among urine NO_x_ and serum NO_x_ (Supplementary Figures 5A–F). The connection between serum nitrate and urine nitrate was most significant (r = 0.593, *p* = 0.000), which may help understand the role of NO metabolites in urine (one non-invasive biological fluid) and blood (invasive biological fluid) in future studies.

Apparently, urine nitrite, urine nitrite/nitrate, serum nitrite, and serum nitrite/nitrate were significantly lower in the ASD-P group than in the Control group or the S-Control group. Though the sample size was limited, urine nitrate, serum nitrite and serum nitrate in mothers of ASD children were consistently lower than that in the female healthy peers. It was the first time that NO metabolites were precisely reported in the parents of children with autism. In order to better understand the role of NO in the ASD-P population, we also provided demographic information for parents in the ASD-P group (43), their children (31), participants in the Control group (43) and participants in the Control group (40) (Supplementary Tables 5, 6).

### Nitrite and Nitrate in the Diet of Parents of Autistic Children

Both endogenous and exogenous nitrogen sources may contribute to NO metabolism in human, so nitrite and nitrate in food were analyzed in order to clarify whether selection of grocery stores impacts content of nitrite and nitrate in food. We made a questionnaire on the diet habits of parents of autistic children, among which spinach, banana and grass carp were the most commonly eaten vegetable, fruit and meat by them. Therefore, we chose these three types of food to represent daily diet of the ASD-P population (Supplementary Table 3). We picked two biggest markets near where the ASD-P group lived, with the geographic distance of about 30 kilometers between the two markets. In order to avoid bias from store to store, each type of food was purchased from three different booths randomly selected inside one market. Sample preparation and analysis was immediately performed after purchase of food to avoid degradation of nitrite and nitrate.

The results with Student's *t*-test showed that there was no significant difference between these two markets in terms of nitrite and nitrate in each type of food (*p* > 0.05) (Supplementary Table 4). Among spinach, banana and grass carp, nitrate in spinach was much higher than in banana, and the latter was higher than in grass carp, which was in accordance with reported in literature (28). Nitrite in banana was barely detectable, and grass carp had lower nitrite level than spinach. Since pH affects stability of nitrite and nitrate, further analysis of pH was performed for these diet reagents, showing that no significant difference between stores, either. In short, store selection didn't contribute to differences of nitrate or nitrite levels in the diet of the ASD-P group.

## Discussion

In this study, we analyzed the levels of urine creatinine, nitrite and nitrate of parents of ASD children for the first time. Lower nitrite, nitrate and nitrite/nitrate were found in this obliterated population compared to the Control group. In addition, mothers had dramatically lower nitrate levels than female participants in the Control, but nitrate in males was similar.

Creatinine is commonly used as an internal standard for adjustment, it also varies with age, sex, ethnicity and time of sampling (29). Here, unadjusted concentrations of nitrite and nitrate (NO_x_) were reported first, and creatinine has been then added as a separate independent variable. We found that male participants had significantly higher creatinine than females in the ASD-P, which was in accordance with the fact that creatinine is usually higher in men than in women (29). Although urinary creatinine level is also closely related to kidney function and psychiatric disorders and depression, anxiety and cognitive disorders are common in patients with kidney diseases (30, 31), creatinine levels between ASD-P and the Control were comparable among participants in this study. Therefore, creatinine was not considered as a biochemical indicator of psychological or physical problems caused by kidney function disorders among parents of autistic children.

NO has been recognized as an independent biomarker of cardiovascular diseases (7). It has a Janus face, serving as both a contributor and scavenger of oxidative stress, depending on its source, concentration and production timing (32). Lower nitrate or nitrite in blood was closely correlated with hypertension and endothelial dysfunction (33). As for the ASD-P group in this study, significantly lower nitrite than healthy controls in the same age range suggests that these parents might be experiencing cardiovascular disorder. These parents had been the major caregiver and attended intervention therapy of their children for at least 6 months. According to the guideline of Applied Behavior Analysis (ABA) and at Huangshi Maternity and Child Health Hospital, ASD children needed to spend at least 35 h per week for training in hospital, plus extra homework for their parents. Besides overwhelming caring tasks, these families were also undertaking extra financial burden due to training and therapy costs, which was ~44,000 RMB (around $6,200) annually. Compared to reported average annual household income of 30,733 RMB (around $4, 300) at Wuhan in 2019 (34), the financial stress for these families was not negligible. It has been known that chronic caregiving and socioeconomic stress predicts the occurrence of cardiovascular diseases among human beings (35).

Furthermore, these stressors can also cause psychological disorders, such as anxiety and depression, which was suggested closely related to NO metabolism (36). Long-term stress damages plasticity of synapse and accelerates death of neurons, which can lead to a variety of neurobiological and psychological problems including memory loss and depressive symptoms (37). Chronic stress also caused decreased expression and activity of nNOS and lower nitrite in the hippocampus of mice (38). In fact, most families with autistic children had mothers as the full time housewives. The available literature showed that mothers of these children were rather more susceptible to major depression disorder (39–41).

The stress levels in parents of ASD children are mainly impacted by these factors: severity of symptoms and behavioral problems of children with ASD, support from society, and parent's personality. One study showed that the severity of the core features of autism was positively correlated with parental stress and mother's psychopathological symptoms (42). Support from professions, families and society (especially friends) was reported to negatively correlate with parental pressure (43, 44). On the other hand, parents experienced less stress if they were more optimistic about life, had stronger personality or held a better acceptable attitude toward their children's disorder (45). Further systematic measurement of parents' stress levels and children's symptoms are needed for sure.

Other than potential endogenous causes for low NO_x_, diet choice may contribute to this difference among parents of autistic children as well, though no significant difference was found from different booths in the two markets near where the ASD-P group lived. Missing detailed information of ASD-P's daily three meals is one limit in this study, which is in our future research plan. In fact, studies in literature and our previous work showed that diet didn't impact nitrite and nitrate in healthy adults (23, 46). It would be more convincing if three meals and multiple time-point urine samples are collected and analyzed on the same day. Therefore, a systematic investigation of exogenous diet sources is needed to evaluate their contribution to urine NO_x_ among the ASD-P population. In addition, mothers appear to be at greater health risks from factors such as lower nitrate. Many autistic children had nutritionally poor diet, due to selective eating, preference for highly processed food, less intake of fruits and vegetables (47). Since parental food habits and feeding strategies greatly influenced children's eating behavior (48), poor diet habit among ASD children may reflect bad food choices for the whole family. These phenomena definitely deserve further investigation of NO signaling and metabolism among the ASD family population.

## Conclusion

Due to lack of understanding of autism worldwide, the autistic family population has been historically underrepresented. Apparently, health condition of parents of autistic children has been barely reported by researchers and clinicians. NOx, a stable metabolite of NO, is closely related to health status of human beings. In this study, urine nitrite and nitrate concentration were studied systematically in parents of autistic children for the first time. The levels of urine ${\text{NO}}_{2}^{-}$, ${\text{NO}}_{3}^{-}$, and ${\text{NO}}_{2}^{-}$/${\text{NO}}_{3}^{-}$ in parents of autistic children were 0.2068 ± 0.2277 μM (arranged from 0.0151 to 1.0173 μM), 3.142 ± 2.144 μM (arranged from 0.6615 to 10.4814 μM), and 0.0789 ± 0.0703(arranged from 0.0033 to 0.3411 μM), which were significantly lower than their peers. In addition, mothers of ASD children had significantly lower nitrate (0.1546 ± 0.1432 μM) than other females (0.6621 ± 0.4624). The significantly lower of NOx in ASD-P indicated potential psychological or physical disorder among them. Through uncovering the levels of NO and its role in these parents, i.e., identifying NO as a potential biomarker, it may help shift future research focus to this specific group of parents. It will surely benefit prevention, diagnosis and therapy for autistic children as well.

## Data Availability Statement

The datasets presented in this article are not readily available because In order to protect the privacy of participants, general information of them are not available to the public. Requests to access the datasets should be directed to Jun Wang, jun_wang@hbut.edu.cn.

## Ethics Statement

The studies involving human participants were reviewed and approved by the Ethics Committee of Huangshi Maternity and Children's Health Hospital (case number: 2017-KF-001). The patients/participants provided their written informed consent to participate in this study.

## Author Contributions

LY, KC, FM, HJ, FX, YL, SL, and JW: conceptualization. LY, KC, FM, FX, and YL: data collection. LY, FX, YL, LB, KP, and JW: formal analysis. FX, YL, LB, KP, and JW: methodology. LY, SL, and JW: software. LY, KC, FM, FX, YL, SL, and JW: writing—original draft preparation. LY, FM, HJ, FX, YL, SL, and JW: writing—review and editing. All authors have read and agreed to the published version of the manuscript.

## Conflict of Interest

The authors declare that the research was conducted in the absence of any commercial or financial relationships that could be construed as a potential conflict of interest.
