# Supplementary material for: Urine Nitric Oxide Is Lower in Parents of Autistic Children
Source: Front Psychiatry. 2021 May 21;12:607191. doi: 10.3389/fpsyt.2021.607191 (PMC8175662; doi:10.3389/fpsyt.2021.607191)
Supplement: Supplementary file 1 [file Data_Sheet_1.PDF]

Supplementary Information of

Urine nitric oxide is lower in parents of autistic children

List of supplementary figures and Tables:

Content	Page
**Supplementary Figure S1.** Standard curves of nitrite and nitrate.	2
**Supplementary Figure S2.** Levels of serum nitrite, nitrate, nitrite/nitrate in the parents of autistic children (ASD-P) and the healthy adults without autistic descendants (S-Control).	3
**Supplementary Figure S3.** The interaction effect of group and sex on nitrate, nitrate/creatinine and nitrite.	4
**Supplementary Figure S4.** Examination of serum nitrite, nitrate and nitrite/nitrate *versus* age.	5
**Supplementary Figure S5.** Examination of serum NO_X and urine NO_X in the ASD-P group.	6
**Supplementary Table S1.** Urinary creatinine, nitrite, nitrate, nitrite/creatinine, nitrate/creatinine, and nitrite/nitrate in each group and subgroup.	7
**Supplementary Table S2**. Serum nitrite, nitrate, and nitrite/nitrate in each group and subgroup.	8
**Supplementary Table S3**. A survey of dietary habits of parents of autistic children.	9
**Supplementary Table S4**. Levels of nitrite, nitrate, and pH of three types of most common food in the diet of parents of autistic children.	9
**Supplementary Table S5**. Demographic information of the ASD-P group and related control population who provided urine samples, as well the S-control group who provided serum samples.	9
**Supplementary Table S6.** Demographic information of autistic children.	10

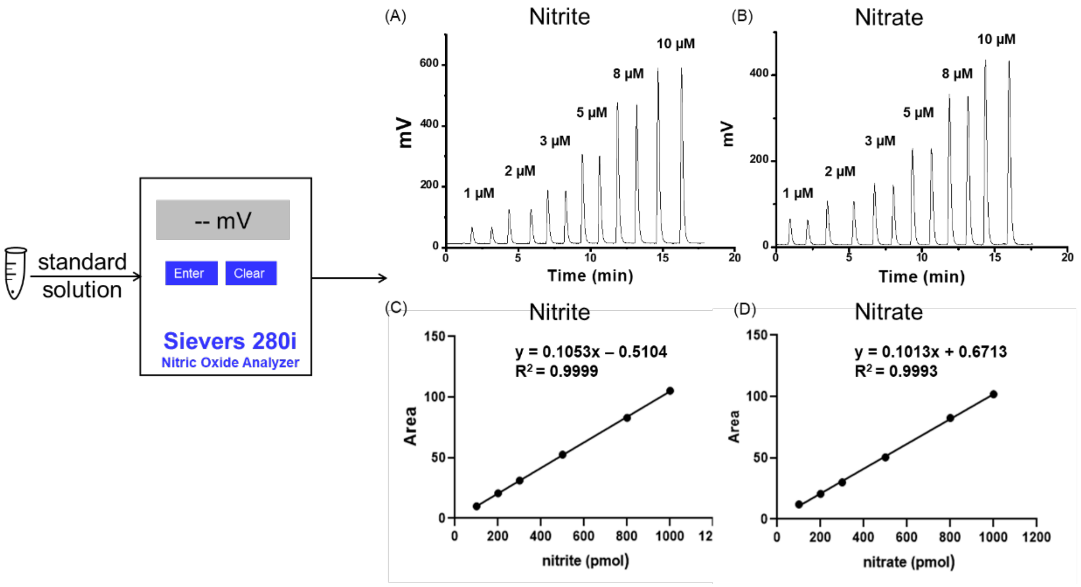

Supplementary Figure S1. Standard curves of nitrite and nitrate. (A, C) The chemiluminescence diagram of nitrite signal, and the fitting curve of standard nitrite solutions. (B, D) The chemiluminescence diagram of nitrate signal, and the fitting curve of standard nitrate solutions.

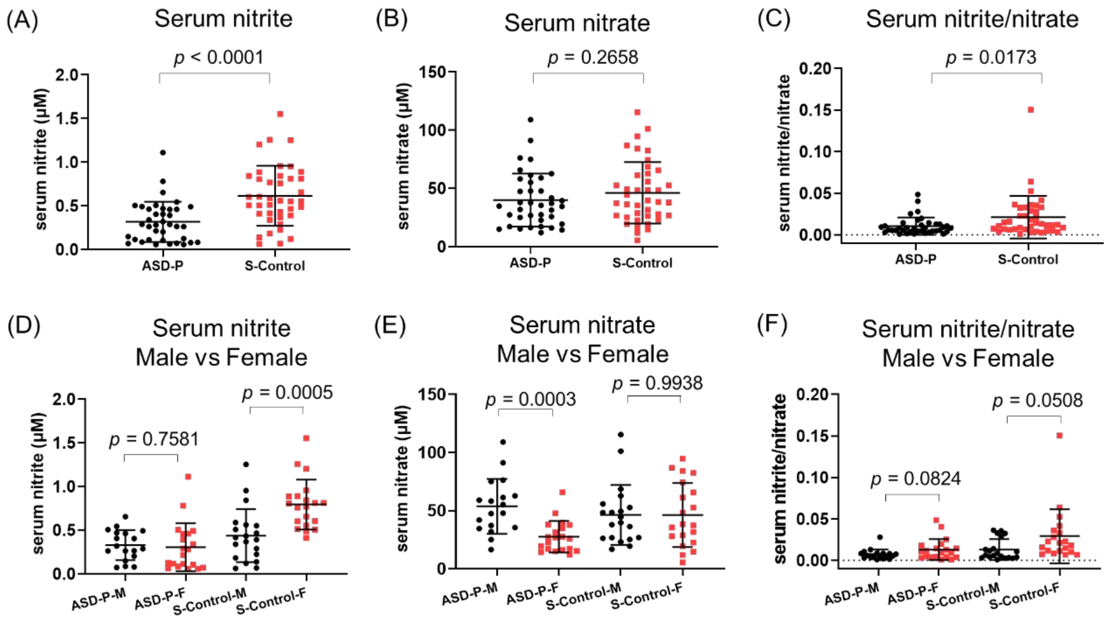

Supplementary Figure S2. Levels of serum nitrite, nitrate, nitrite/nitrate in the parents of autistic children (ASD-P) and the healthy adults without autistic descendants (S-Control). (A) nitrite concentrations in two groups. (B) nitrate concentrations in two groups. (C) nitrite/nitrate levels in two groups. (D) nitrite concentrations for male and female participants. (E) nitrate concentrations for male and female participants. (F) nitrite/nitrate concentrations for male and female participants.

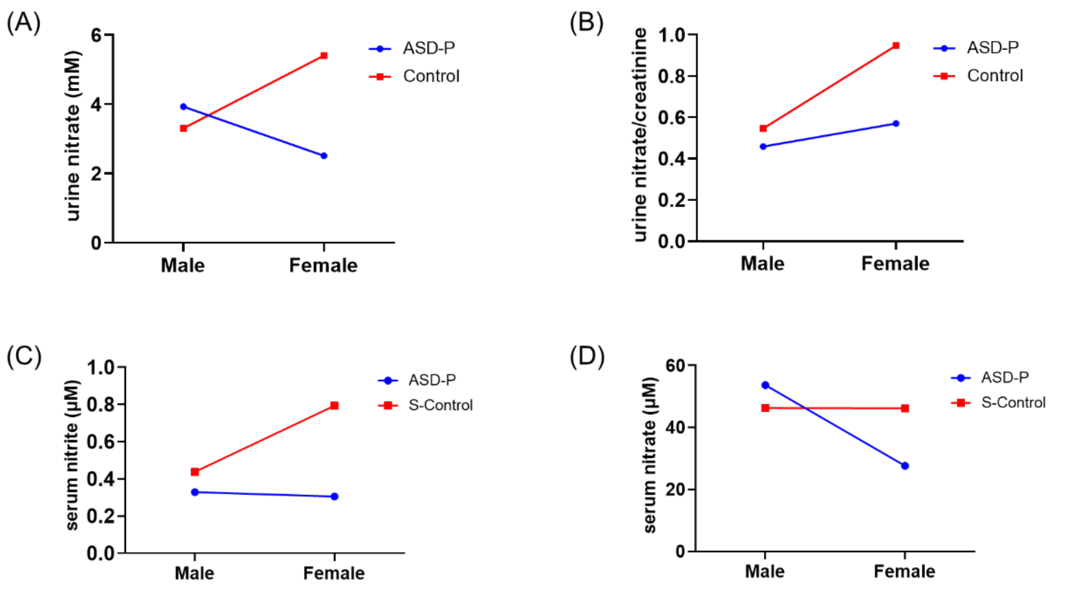

Supplementary Figure S3. The interaction effect of group and sex on nitrate, nitrate/creatinine and nitrite. (A) The interaction effect of group and sex on urine nitrate. (B) The interaction effect of group and sex on urine nitrate/creatinine. (C) The interaction effect of group and sex on serum nitrite. (D) The interaction effect of group and sex on serum nitrate.

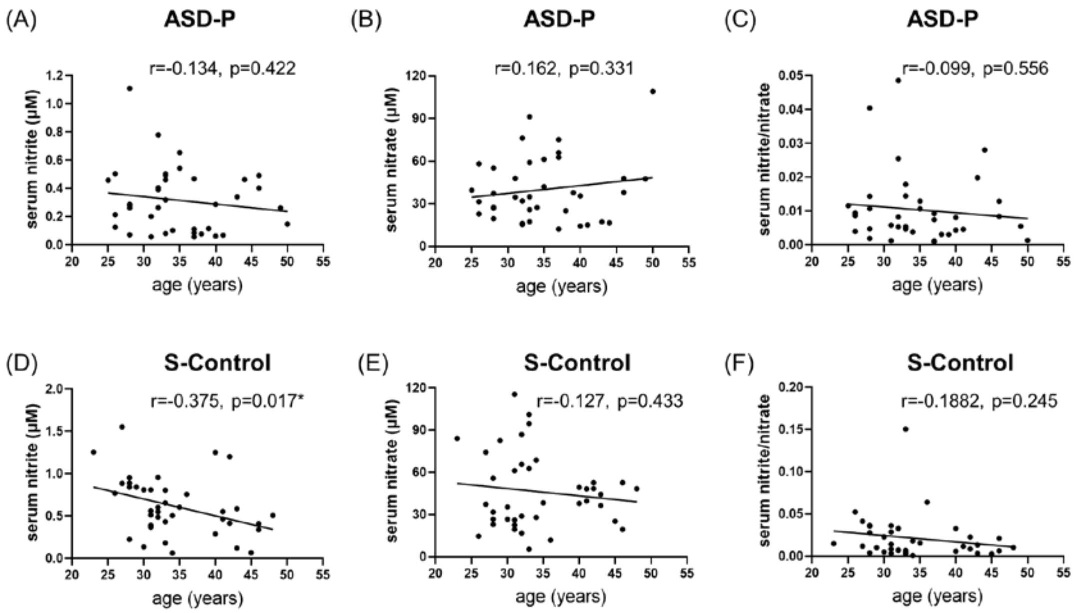

Supplementary Figure S4. Examination of serum nitrite, nitrate and nitrite/nitrate *versus* age. (A) serum nitrite and age in the ASD-P group, p = 0.422. (B) serum nitrate and age in the ASD-P group, p = 0.331. (C) serum nitrite/nitrate and age in the ASD-P group, p = 0.556. (D) serum nitrite and age in the S-Control group, p = 0.017. (E) serum nitrate and age in the S-Control group, p = 0.433. (F) serum nitrite/nitrate and age in the S-Control group, p = 0.245.

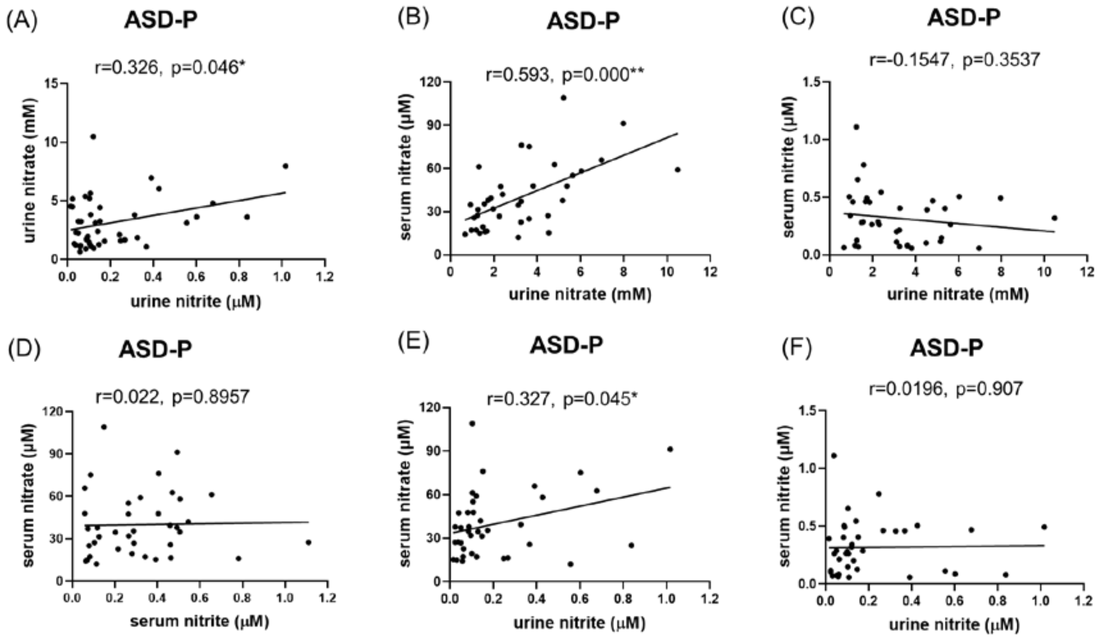

Supplementary Figure S5. Examination of serum NO_X and urine NO_X in the ASD-P group. (A) urine nitrite and urine nitrate in the ASD-P group, p = 0.046. (B) urine nitrate and serum nitrate in the ASD-P group, p =0.000. (C) urine nitrate and serum nitrite in the ASD-P group, p =0.3537. (D) serum nitrite and serum nitrate in the ASD-P group, p = 0.8957. (E) urine nitrite and serum nitrate in the ASD-P group, p =0.045. (F) urine nitrite and serum nitrite in the ASD-P group, p =0.907.

Supplementary Table S1. Urinary creatinine, nitrite, nitrate, nitrite/creatinine, nitrate/creatinine, and nitrite/nitrate in each group and subgroup.

Groups \ Values	CREA(mM)	NO_2^-(μM)	NO_3^-(mM)	NO_2^-/CREA (*10^{-3})	NO_3^-/CREA	NO_2^-/ NO_3^- (*10^{-3})
ASD-P	8.352±5.513	0.2068±0.2277	3.142±2.144	0.0383±0.0497	0.5208±0.4755	0.0789±0.0703
(n=43)	(1.15-21.87)	(0.0151-1.0173)	(0.6615-10.4814)	(0.0022-0.21)	(0.0751-2.0139)	(0.0033-0.3411)
Control	7.067±4.487	0.6518±0.4355	4.627±3.847	0.1564±0.2114	0.7986±0.7876	0.3476±0.6208
(n=43)	(0.51-17.55)	(0.155-2.4577)	(0.1276-16.9125)	(0.0212-1.3392)	(0.0425-4.3399)	(0.0317-3.5158)
p-value	0.2394	<0.0001	0.0305	0.0008	0.0518	0.0072
ASD-P -M (n=19)	10.52±5.483	0.2727±0.2942	3.934±2.44	0.0342±0.0389	0.4584±0.3454	0.0754±0.0634
ASD-P -F (n=24)	6.638±5.003	0.1546±0.1432	2.514±1.676	0.0415±0.0574	0.5703±0.5601	0.0817±0.0766
p-value	0.0201	0.1209	0.0294	0.6364	0.4260	0.7732
Control -M (n=16)	6.902±4.447	0.6345±0.3998	3.308±2.763	0.1337±0.1096	0.5466±0.3437	0.3779±0.5204
Control -F (n=27)	7.165±4.592	0.6621±0.4624	5.409±4.218	0.1698±0.2544	0.9479±0.9336	0.3297±0.6822
p-value	0.8550	0.8439	0.0832	0.5232	0.0514	0.8091

CREA : creatinine

Supplementary Table S2. Serum nitrite, nitrate, and nitrite/nitrate in each group and subgroup.

Groups \ Values	NO_2^-(μM)	NO_3^-(μM)	NO_2^-/ NO_3^-
ASD-P (n=38)	0.3167±0.2304 (0.0577-1.111)	39.94±22.88 (12.11-109.1)	0.0104±0.0104 (0.0009-0.0486)
S-Control (n=40)	0.6155±0.3431 (0.0624-1.552)	46.22±26.36 (5.355-115.4)	0.0212±0.0257 (0.0009-0.1506)
p-value	<0.0001	0.2658	0.0173
ASD-P -M (n=18)	0.3291±0.1734	53.65±23.62	0.0074±0.006055
ASD-P -F (n=20)	0.3056±0.2760	27.60±13.49	0.0131±0.01267
p-value	0.7581	0.0003	0.0824
S-Control -M (n=20)	0.4375±0.3049	46.26±25.81	0.01318±0.01253
S-Control -F (n=20)	0.7935±0.2863	46.19±27.56	0.02924±0.03263
p-value	0.0005	0.9938	0.0508

Supplementary Table S3. A survey of dietary habits of parents of autistic children.

Vegetables	spinach	lettuce	cabbage	celery	rape	cucumber
N (%)	31 (80.34)	23 (60.87)	19 (50)	7 (39.13)	13 (34.78)	13 (34.78)
Fruits	banana	orange	peach	apple	pineapple	grape
N (%)	24 (65.22)	22 (58.7)	22 (58.7)	18 (47.83)	14 (36.96)	12 (32.61)
Meat	grass carp	crucian	sole fish	carp	catfish	black carp
N (%)	26 (69.57)	24 (63.04)	21 (56.52)	17 (45.65)	13 (34.78)	12 (30.43)

Supplementary Table S4. Levels of nitrite, nitrate, and pH of three types of most common food in the diet of parents of autistic children.

Specimen	Nitrate (mg/kg)	*p*-value	Nitrite (mg/kg)	*p*-value	pH	*p*-value
M1-spinach	561.89-1224.22	0.1311	0.60-3.38	0.5975	6.32-6.41	0.5579
M2-spinach	1075.40-2118.91		0.34-2.51		6.34-6.44	
M1-banana	63.16-163.50	0.4630	ND-0.04	0.2537	4.75-5.19	0.4628
M2-banana	35.85-114.25		ND-0.01		4.76-4.96	
M1-grass carp	0.92-2.19	0.0771	0.07-0.24	0.3730	6.44-6.76	0.6212
M2-grass carp	2.28-2.86		0.05-0.36		6.57-6.70	

M1: market 1
M2: market 2
ND: not detected

Supplementary Table S5. Demographic information of the ASD-P group and related control population who provided urine samples, as well the S-control group who provided serum samples.

sample		ASD-P, N (%)	Control, N (%)
urine sample	Gender		
	Male	19 (44.2)	16 (37.2)
	Female	24 (55.8)	27 (62.8)
	Age (years)		
	< 30	9 (20.9)	9 (20.9)
	30-39.99	26(60.5)	11 (25.6)
	40-50	8 (18.6)	23 (53.5)
sample		ASD-P, N (%)	S-Control, N (%)
serum sample	Gender		
	Male	18 (47.4)	20 (50.0)
	Female	20 (52.6)	20 (50.0)
	Age (years)		
	< 30	9 (23.7)	11 (27.5)
	30-39.99	21 (55.3)	17 (42.5)
	40-50	8 (21.0)	12 (30.0)

Supplementary Table S6. Demographic information of autistic children.

ASD	Data
Male/Female	28/3
Age (mean±SD, years)	4.829±1.663
Gestational age (mean±SD, weeks)	39.12±2.718
Birth weight (mean±SD, kg)	3.373±0.4642
Height (mean±SD, cm)	103.0±21.57
Weight (mean±SD, kg)	21.6±15.15
